# Sperm-borne mRNAs: potential roles in zygote genome activation and epigenetic inheritance

**DOI:** 10.1098/rsob.240321

**Published:** 2025-03-26

**Authors:** Betina González, Candela Rocío González

**Affiliations:** ^1^ Instituto de Investigaciones Farmacológicas, Buenos Aires, Argentina

**Keywords:** sperm, mRNA, transcriptome, embryo development, epigenetic

## Abstract

It is well accepted that sperm carry an RNA cargo with functions in early embryo development. However, most research has focused on the role of small RNAs, such as microRNAs, transfer RNAs and long non-coding RNAs, while protein-coding messenger RNAs (mRNAs) received less attention, even though they represent a substantial amount of the sperm RNA pool. Here, we curated mouse transcriptomic data from mature sperm and selected the most abundant mRNAs (above the 0.7 quantile). The obtained gene list was further filtered using two criteria: (i) mRNAs that are statistically higher in the one-cell embryo compared to the MII oocyte transcriptome, indicative of paternal mRNA contribution after fertilization; and (ii) mRNAs that are found bound to ribosomes in the one-cell embryo, indicative of positive translation in the zygote translatome. Our analysis identified 94 genes that form networks functionally involved in epigenetic chromatin organization, gene expression, RNA processing and translation during zygote genome activation. These findings underscore the significant role of sperm-borne mRNAs in early embryonic development and epigenetic inheritance, highlighting the need for further research to fully understand their functions.

**Figure:**
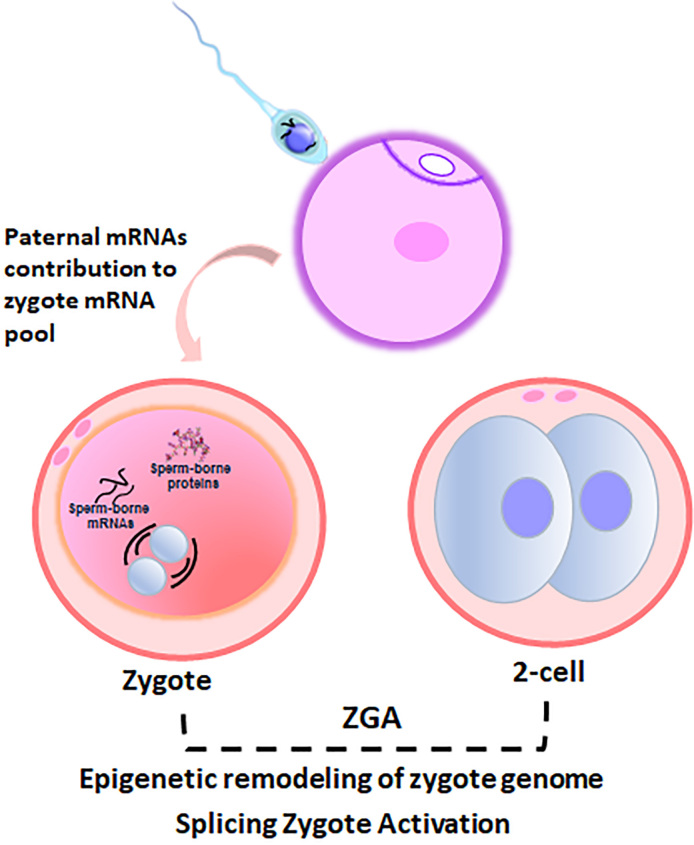

## 1. Background

In recent years, the role of RNAs carried by spermatozoa has gained increased attention in reproductive biology due to its potential implications in fertilization and early embryonic development. Sperm cells, conventionally viewed as simple vehicles for delivering paternal DNA to the oocyte, are now recognized as intricate carriers of a diverse array of RNAs, including microRNA (miRNA), transfer RNA (tRNA), long non-coding RNA (lncRNA) and messenger RNA (mRNA) [1,2]. Recent studies in genomics and transcriptomics have identified thousands of distinct RNAs in sperm, suggesting active transcription and mRNA processing, as well as transcriptional potential in certain chromosomal regions due to histone retention [1,3–5]. Sperm-borne RNAs probably originate during spermatogenesis, but many RNAs are transmitted to mature sperm by extracellular vesicles from the epididymis [6,7]. This diverse RNA repertoire encompasses genes associated with a wide range of functions, including chromatin remodelling, DNA repair, cell signalling and metabolism [5,8,9].

The content of sperm RNAs is not static but can be dynamically influenced by several factors, including environmental exposures, paternal lifestyle and epigenetic modifications [10]. Studies have demonstrated that environmental stressors can alter the expression profile of sperm-borne RNAs, potentially affecting offspring health and development [11–14]. Additionally, epigenetic modifications including DNA methylation and histone post-translational modifications may contribute to the regulation of RNA expression in sperm, further shaping the paternal contribution to early embryogenesis [1,15,16]. Upon sperm entry into the oocyte, these RNAs are believed to play crucial roles in initiating the molecular events related to the activation of key developmental pathways in the early embryo [17,18]. Furthermore, these transcripts may participate in the establishment of epigenetic marks in the embryo, influencing gene expression patterns and phenotypic outcomes in the offspring [3,18,19].

Despite the growing recognition of sperm-borne RNAs' participation in early embryo development and paternal epigenetic inheritance, most of the studies focused on the short non-coding RNAs, whereas the protein-coding mRNAs received far less attention, even though representing a significant amount of the sperm RNA pool. Here, we report that the most abundant mRNAs in mouse sperm may create protein networks controlling epigenetic chromatin organization and RNA processing, with direct impact on the activation of the zygote genome and the regulation of pluripotency and cell fate pathways in the early embryo.

## 2. Methods

Sperm cell data were processed as described previously [18]. Briefly, mature sperm transcriptomic data were retrieved from GSE81216 (total sperm and sperm head C57BL/6 wild-type samples), GSE88732 (total sperm C57BL/6 wild-type samples) and E-MTAB-5834 (total sperm C57BL/6J control samples). We selected studies that performed RNA-Seq of large RNAs or sncRNAs, where the RNA extraction methods for these polyadenylated RNAs efficiently capture the mRNA population. To harmonize the sperm data generated across different laboratories, minimize inter-study variability and enhance the accuracy of mRNA-enrichment detection within each dataset, we reprocessed the raw data uniformly using the same bioinformatic pipeline and normalized the data using the percentile rank conversion method to ensure comparability and consistency across studies. Sperm FASTQ files were mapped with STAR and GRCm38 (MM10, gencode.vM29.annotation), and counting was performed with featureCounts. Counts were converted to reads per kilobase of exon per million mapped reads (RPKM) using the mean gene length extracted from the gencode.vM29 file. The sperm RPKMs obtained for the three selected datasets were converted to percentile rank, and the sperm mean percentile rank was calculated. We selected genes with a mean percentile rank of >0.7. This threshold was selected because it ensured consistency across the datasets, as demonstrated by a reduced standard deviation. Furthermore, genes meeting this threshold consistently showed non-zero values in all datasets, indicating reliable detection across the selected studies. This approach effectively minimized variability and enhanced confidence in the robustness of the identified gene set, ensuring its suitability for further analysis. MII oocytes and one-cell embryos transcriptomic data were obtained from GSE169632 [20] and analysed with the DESeq2 package to select genes significantly upregulated in the zygote (log2FC ˃ 0.5, *p*_adj_ ˂ 0.05). Data from one-cell embryo ribosome-bound RNA RPKMs were also obtained from GSE169632. The embryo translational efficiency (TE) was calculated as Ribo-seq RPKM/total RPKM. Network analysis and functional enrichment were performed at the STRING platform (https://string-db.org/). Raw data and bioinformatic analysis can be found in https://github.com/Gonzalez-Lab/spermRNAs.

## 3. Results

### 3.1. Functional characterization of the most abundant mRNAs in the mature sperm

**Figure 1.**
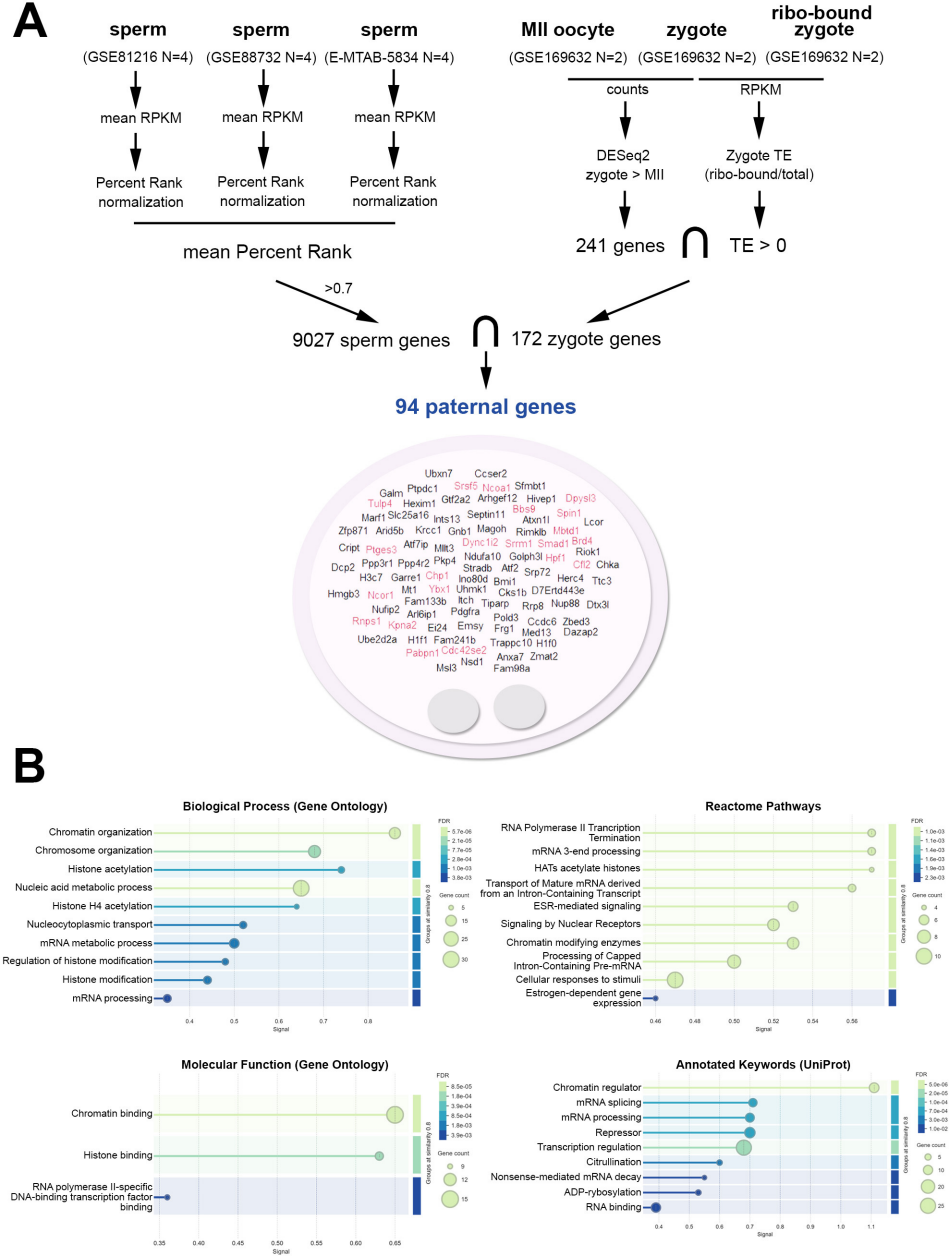
Selection of sperm-borne mRNAs and functional enrichment analysis. (A) Gene analysis pipeline. Mature sperm transcriptomic data (RPKM) was converted to mean percentile rank, and genes above the 0.7 percentile were selected as highly enriched sperm-borne mRNAs. These genes were filtered (i) with the gene list of significantly higher mRNA levels in zygotes compared with MII oocytes (DESeq2, GSE169632), indicative of paternal contribution, and (ii) with the gene list of mRNAs bound to ribosomes (translatome, GSE169632) in the zygote, presenting positive translation efficiency. The word cloud plot shows the 94 sperm-borne mRNAs obtained, and in red lettering, those present in the ‘embryo’ and ‘embryonic structure’ signatures (false discovery rate (FDR) ˂ 0.05). (B) Results of the functional enrichment analysis with respect to Gene Ontology (biological processes and molecular functions), reactome pathways and annotated keywords (UniProt). Circle size indicates gene count, and the colour scale represents the FDR, with darker colours indicating higher significance.

To characterize the sperm mRNA pool that could be specifically delivered and translated in the zygote, we used transcriptomic data from mature sperm, meiosis II (MII) oocytes and zygotes (figure 1A). We focused on mRNAs above the 0.7 quantile and further filtered for those significantly enriched in the zygote compared with MII oocytes, indicating paternal contribution. We retained mRNAs with positive TE in the zygote, identifying 94 candidates potentially involved in the one-cell embryo's first cellular processes. Figure 1A summarizes the data processing pipeline and shows a word cloud plot of the obtained mRNAs, where red lettering shows genes that enriched significantly in the ‘embryo’ (false discovery rate (FDR) = 0.0072) and ‘embryonic structure’ (FDR = 0.0142) signatures (full details of the functional enrichment results performed at STRING are listed in electronic supplementary material, table S1). The functional enrichment analysis based on STRING data of the proteins encoded by the 94 sperm-borne mRNAs is shown in figure 1B. Notably, the analysis reveals significant enrichment in chromatin and chromosome organization and binding, histone acetylation, histone acetyltransferases, and chromatin-modifying enzymes. The analysis also highlights significant processes, including RNA metabolic processes, RNA polymerase II transcription termination, mRNA processing and splicing.

### 3.2. Identification of functional networks involved in chromatin remodelling and RNA regulation

**Figure 2.**
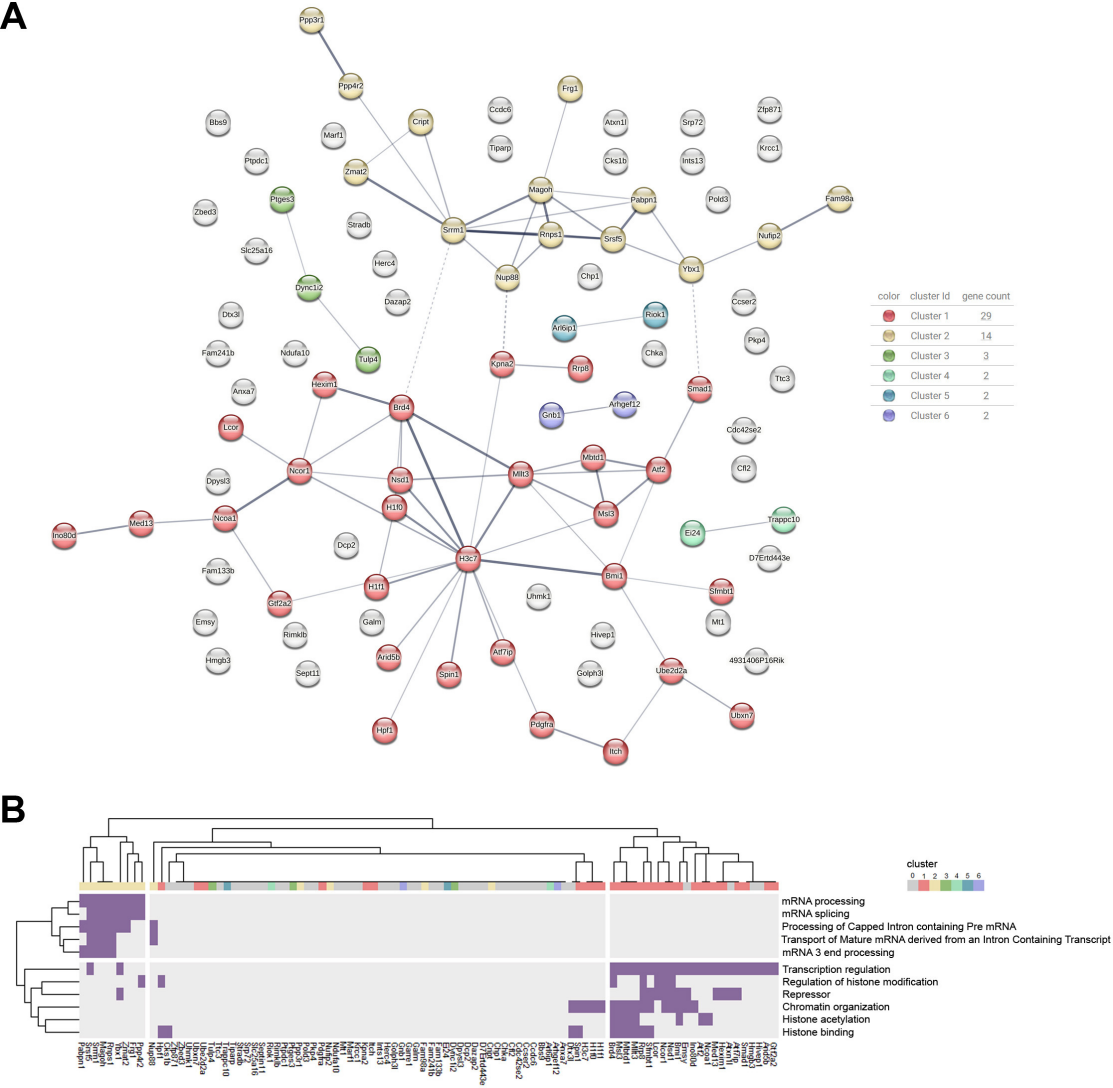
Protein network analysis of sperm-borne mRNAs. (A) Protein network analysis of the 94 sperm-borne mRNAs (protein–protein interaction enrichment *p*‐value: 0.000237) performed at the STRING platform. The lines represent protein interactions, with line thickness indicating the confidence level of the supporting evidence. (B) Heatmap and hierarchical clustering of the 94 sperm-borne mRNAs that were matched (violet) to functional terms showing a strength ˃ 0.7 and FDR < 0.05.

Network analysis performed on the 94 genes showed significant protein–protein interaction (PPI) enrichment (*p* = 0.000237) (figure 2A). We found six distinct clusters (*k*-means) of closely connected proteins potentially involved in similar biological processes or functional pathways, with two prominent and interconnected red and yellow clusters. The remaining four smaller clusters and genes outside the main network did not show any functional terms significantly enriched. Figure 2B shows the most robust terms obtained in the functional enrichment analysis (strength > 0.7, FDR < 0.05) and the genes belonging to each signature. Clustering analysis of genes and terms shows a functional segregation between the clusters, where most of the genes in the red cluster belong to the signatures associated with chromatin organization and transcription regulation, whereas most of the genes in the yellow cluster belong to the signatures involved in mRNA processing (figure 2B).

The red cluster is centred around the core nucleosome component histone 3.1 (H3C7), with the linker histones H1F1 and H1F0 directly interacting with H3. As shown in figure 2B, this cluster also comprises proteins involved in histone modifications and binding, such as NSD1 (Nuclear Receptor Binding SET Domain Protein 1), writer of H3K36me2 and the readers MLLT3 (Super Elongation Complex Subunit), which recognizes crotonylated H3 to mark active promoters and enhancers, and SPIN1 (Spindlin 1), which recognizes H3K4me3/R8me2a and non-canonical bivalent marks. Other proteins in the cluster are specifically associated with histone acetylation, including the acetyltransferase MSL3 (MSL Complex Subunit 3), which mediates H4K16ac, and the acetylation readers MBTD1 (Mbt Domain Containing 1) and BRD4 (Bromodomain Containing 4). Within this cluster, we also found proteins involved in activating transcription through acetylation, such as NCOA1 (Nuclear Receptor Coactivator 1), MED13 (Mediator Complex Subunit 13), GTF2A2 and ATF2, among others. Additionally, we identified the Repressor signature, associated with factors such as NCOR1 (Nuclear Receptor Co-Repressor 1), BMI1 (BMI1 Proto-Oncogene, Polycomb Ring Finger) and Scm Like With Four Mbt Domains 1. The red cluster showed functional interconnections with the yellow cluster, mainly associated with RNA transport, processing and splicing, and key genes related to these processes, such as Y-Box Binding Protein 1, Serine and Arginine Repetitive Matrix 1, Mago Homolog, exon junction complex subunit and Cysteine-Rich PDZ-Binding Protein, among others.

## 4. Discussion

In recent years, the idea that sperm-borne mRNAs are simply remnants of spermatogenesis has evolved, especially with the observation that selective mRNA retention occurs while large amounts of ribosomal RNA are degraded. Although mature sperm contains several mRNAs, most studies suggest they become more relevant after fertilization [2]. Despite challenges in isolating sperm-borne RNAs and obtaining high-quality embryonic cells, evidence supports that both coding and non-coding paternal RNAs play functional roles in early development and epigenetic inheritance [3,18,21,22]. We previously showed that sperm transport epigenetic enzyme mRNAs that are translated in the zygote and may carry transgenerational epigenetic information [18]. Here, we identified a distinct set of sperm-borne mRNAs involved in protein networks for activation of the zygotic genome, including processes like chromatin organization, mRNA metabolism and gene expression.

After fertilization, transcriptional control is transferred to the zygote through the maternal-to-zygotic transition (MZT), a process where maternal products are degraded in coordination with specific mRNAs translation that will enable the zygotic genome activation (ZGA) [23,24]. In line with this, we found a cluster of sperm-borne mRNAs involved in mRNA processing and splicing, implying that the sperm could transport specific instructions to modulate these processes in the one-cell embryo. This aligns with the essential role of zygotic splicing activation (ZSA), where precise isoform expression complements transcriptional activation during MZT to support preimplantation development [25]. The shift between MZT to ZGA underscores the crucial role of epigenetic modifications, including their genome-wide establishment and reestablishment [26–29]. Despite extensive reprogramming, some regions remain epigenetically distinct, with histone modifications and variants crucial for gene expression and precise control over ZGA [22,30–34]. Here, we found that sperm-borne mRNAs, encoding key epigenetic regulators such as enzymes and chromatin-associated factors, play a central role in shaping the paternal contribution to the early embryo through the establishment of the chromatin landscape required for ZGA. We observed that H3.1 emerges as a central gene within the main cluster, and current findings emphasize that paternal chromatin remodelling mediated by H3 is essential for activating the paternal genome during embryogenesis [29]. During ZGA, the histone modifications H3K27me3 and H3K27ac shape the transcription of critical genes [29], with H3K27me3 repressing alternative cell fate genes to maintain cellular identity [30,31], while H3K27ac promotes transcriptional activity as the zygotic genome activates [35]. Importantly, we found BRD4, which has been recently described to trigger premature ZGA and increase H3K27ac levels, indicating that BRD4 enhances transcriptional competency during the MZT [36]. Furthermore, BRD4 regulates Nanog expression [36] and is crucial for the transcriptional activation of the X chromosome, which occurs during major ZGA and co-regulates X-linked expression together with imprinted X chromosome inactivation [37]. We also found paternal contribution of NSD1, which modulates the deposition of the repressive mark H3K27me3, balancing active and repressed states of genes during early development [38]. NSD1 also deposits H3K36me2 and recruits HDAC1 at active enhancers to prevent further gene activation, with reduced H3K36me2 linked to increased H3K27ac and enhanced gene expression related to mesoderm differentiation [38]. Additionally, we identified SPIN1, which recognizes active enhancer/promoter H3K4me3 and regulates stem cell potency, lineage determination and ZGA genes, being essential for the gamete-to-embryo transition [29,39]. We identified two histone acetylation factors that contribute to chromosome stability, with MBTD1 playing a role in DNA repair [40] and the MSL3 complex maintaining homeostatic levels of H4K16ac, while regulating Hox clusters [41,42]. In addition, we found the PRC1 component BMI1, which regulates stem cell proliferation and self-renewal through histone mark H2AK119ub1 [43], which is believed to contribute to resistance against transcriptional reprogramming, as loss of H2AK119ub1 results in premature activation of developmental genes during ZGA [44]. Finally, we identified important transcription factors and co-activators within this network: NCOR1, NCOA1, MED13 and GTF2A2. MED13 regulates ZGA and is essential for post-implantation development, facilitating zygote reprogramming into a totipotent embryo [45]. NCOR1 is involved in embryonic stem cell fate decisions [46,47], somitogenesis [48] and neural stem cell differentiation [49–52]. GTF2A2 maintains pluripotency and ensures proper progression of embryonic development [53], while NCOA1 plays key roles in placental development and embryo survival [54].

Although parental genomes undergo extensive epigenetic reprogramming in the zygote, it remains unknown whether the parental genomes play distinct roles during ZGA. Interestingly, a recent study found that the major ZGA seems to be first initiated from the paternal genome in human embryos [34]. While previous research primarily focused on the perturbation of sperm non-coding RNAs and their immediate effects on early embryos, our findings provide substantial evidence that mature sperm cells carry a unique set of paternal mRNAs crucial for MZT and ZGA after fertilization. The presence of these transcripts in the translatome of the one-cell embryo suggests their involvement in initiating transcriptional control post-fertilization. While this study is based on *in silico* analysis, it underscores the need for future experimental research to validate the translation of sperm-borne mRNAs into proteins in the zygote, which is crucial for understanding their contribution to the proteomic profile of the fertilized egg and their roles in early embryonic development and epigenetic inheritance.

## Data Availability

The datasets supporting this article have been uploaded to GitHub [55]. Supplementary material is available online [56].
